# Cryoelectron microscopy structures of a human neutralizing antibody bound to MERS-CoV spike glycoprotein

**DOI:** 10.3389/fmicb.2022.988298

**Published:** 2022-09-28

**Authors:** Shuyuan Zhang, Wenxv Jia, Jianwei Zeng, Mingxi Li, Ziyi Wang, Haixia Zhou, Linqi Zhang, Xinquan Wang

**Affiliations:** ^1^The Ministry of Education Key Laboratory of Protein Science, Beijing Advanced Innovation Center for Structural Biology, Beijing Frontier Research Center for Biological Structure, School of Life Sciences, Tsinghua University, Beijing, China; ^2^Comprehensive AIDS Research Center and Beijing Advanced Innovation Center for Structural Biology, School of Medicine, Tsinghua University, Beijing, China; ^3^NexVac Research Center, Tsinghua University, Beijing, China

**Keywords:** neutralizing antibody, MERS-CoV, spike glycoprotein, cryo-EM structures, neutralization mechanism

## Abstract

Neutralizing monoclonal antibodies (mAbs) against highly pathogenic coronaviruses represent promising candidates for clinical intervention. Here, we isolated a potent neutralizing monoclonal antibody, MERS-S41, from a yeast displayed scFv library using the S protein as a bait. To uncover the neutralization mechanism, we determined structures of MERS-S41 Fab in complex with the trimeric spike glycoprotein by cryoelectron microscopy (cryo-EM). We observed four distinct classes of the complex structure, which showed that the MERS-S41 Fab bound to the “up” receptor binding domain (RBD) with full saturation and also bound to an accessible partially lifted “down” RBD, providing a structural basis for understanding how mAbs bind to trimeric spike glycoproteins. Structure analysis of the epitope and cell surface staining assays demonstrated that virus entry is blocked predominantly by direct competition with the host receptor, dipeptidyl peptidase-4 (DPP4).

## Introduction

The outbreak of coronavirus disease 2019 (COVID-19), caused by the severe acute respiratory syndrome coronavirus 2 (SARS-CoV-2), has been declared by the World Health Organization (WHO) as a global health emergency that, at the time of writing, has been responsible for more than 6 million deaths.^1^ Unfortunately, it is just the latest in a line of lethal respiratory diseases spread by coronaviruses to cause worldwide epidemics. SARS-CoV-1 emerged in 2002, resulting in 8,000 infections and nearly 800 deaths in 37 countries (Peiris et al., 2003). In 2012, Middle East respiratory syndrome coronavirus (MERS-CoV), emerged in the Arabian peninsula and caused numerous outbreaks in humans, with a fatality rate of 35%.^2^ MERS-CoV likely originated from bats, with camels functioning as a nature reservoir (Azhar et al., 2014; Mohd et al., 2016). Small clusters of infections without camel exposure in several countries suggested that human-to-human transmission can occur through close contact (Zumla et al., 2015). Due to the high pathogenicity, significant lethality and verified capability of human-to-human transmission, there has been a persistent concern that MERS-CoV could cause a disruptive pandemic.

Coronavirus trimeric spike (S) glycoproteins mediate viral entry. The MERS-CoV S protein undergoes protease cleavage into two subunits (Millet and Whittaker, 2014), leading to non-covalently associated S1 and S2 subunits, whereby a trimer of S1 sits atop a trimer of S2 subunits. S1 is responsible for binding to the host receptor, and contains the N-terminal domain (NTD), the receptor binding domain (RBD), and subdomain 1 and 2 (SD1 and SD2). S1 adopts dynamic conformations in cryo-EM structures of prefusion MERS-CoV (Pallesen et al., 2017; Yuan et al., 2017), SARS-CoV (Gui et al., 2017; Yuan et al., 2017), and SARS-CoV-2 (Walls et al., 2020; Wrapp et al., 2020), wherein RBDs adopt either a “down” conformation that buries the receptor-binding surface, or an “up” conformation that facilitates binding with host-cell receptors. The binding of S1 RBD to the host receptor DPP4 (Lu et al., 2013; Raj et al., 2013; Wang et al., 2013) likely initiates the fully receptor-binding capable state in which all three RBDs adopt the “up” conformation, resulting in conformational change of S2, which mediates the fusion of the viral and host-cell membranes (Pallesen et al., 2017; Yuan et al., 2017; Walls et al., 2019). As the S protein decorates the viral surface and is vital for its infectivity, it is the main target of neutralizing antibodies. Numerous monoclonal antibodies (mAbs) against the S protein of MERS-CoV have been reported, isolated from single chain fragment variable (scFv) libraries (Jiang et al., 2014; Tang et al., 2014; Ying et al., 2014), generated from immunized animals (Li et al., 2015; Wang et al., 2015; Chen et al., 2017) or based on B cell cloning from convalescent individuals (Corti et al., 2015). Most neutralizing antibodies were isolated using the RBD as a bait. Structural studies have revealed that RBD-targeting mAbs directly or indirectly disrupt the interaction between RBD and DPP4 (Li et al., 2015; Wang et al., 2015, 2018; Ying et al., 2015; Yu et al., 2015; Chen et al., 2017; Niu et al., 2018; Zhang et al., 2018). The trimeric spike glycoprotein has also used as a bait for selection from phage displayed scFv libraries or to inoculate mice. Successful examples are antibody 3B11 isolated from a scFv library (Tang et al., 2014) and mAb 7D10 generated from S protein immunized mice (Zhou et al., 2019). Since the trimeric S protein has the potential to isolate mAbs targeting various sites including the RBD, NTD, S2, or structural elements of the trimer, it could be a more effective antigen to select mAbs than the RBD.

Here, we report the isolation of a potent neutralizing monoclonal antibody, MERS-S41, from a yeast displayed scFv library using the S protein as the bait. To determine the neutralization mechanism and further explore the interactions between MERS-S41 and S protein, we resolved the structures of the trimeric spike glycoprotein in complex with the MERS-S41 Fab by cryoelectron microscopy (cryo-EM). The structures showed that MERS-S41 can bind the MERS-CoV spike with different stoichiometries, in which MERS-S41 Fab was able to bind “up” RBD and partially lifted “down” RBD, whereas MERS-S41 Fab was unable to bind fully “down” RBD. As a result of structure analysis of the epitope and cell surface staining assay, the main neutralization mechanism for MERS-S41 is demonstrated to be direct competition with DPP4.

## Results

### Neutralizing mAb MERS-S41 isolated from non-immune human antibody library

**FIGURE 1:**
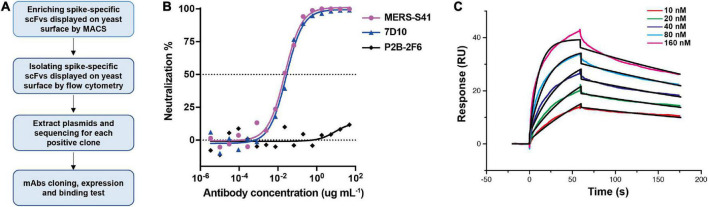
Antibody MERS-S41, isolated from a scFv yeast display library, has potent neutralizing activity. **(A)** The selection flowchart. **(B)** Neutralization of the MERS-S41 IgG against pseudotyped MERS-CoV. Antibody 7D10 IgG targets NTD of MERS-CoV S and P2B-2F6 targets RBD of SARS-CoV-2, as positive and negative controls, respectively. **(C)** Surface plasmon resonance curves showing binding of MERS-CoV spike glycoprotein to immobilized MERS-S41 IgG. Data are shown as different colored lines and the best fit of the data to a 1:1 binding model is shown in black.

To generate neutralizing mAbs against the MERS-CoV glycoprotein spike, we first expressed and purified the ectodomain of the spike as described before (Gui et al., 2017; Supplementary Figure 1). The bait was then used to select antibodies from a non-immune human scFv library displayed on the surface of the yeast, *Saccharomyces cerevisiae* (Jiang et al., 2014). The selection procedure is shown in Figure 1A. We performed two rounds of magnetic bead–activated cell sorting (MACS) followed by three rounds of florescence-activated cell sorting (FACS). Plasmids containing the coding sequences for scFv were then extracted from the sorted yeast population, and proliferated in *Escherichia coli* for sequence analysis.

Among the 119 plasmid sequences analyzed, 13 distinct scFv sequences were identified (Supplementary Table 1). Interestingly, one of the scFv sequences, MERS-S111, was identical to the MERS-27 sequence which we isolated previously using RBD as the bait (Yu et al., 2015). Subsequently, we grafted the scFv sequences onto constant region sequences to generate full-length IgG1s which possess better thermal stability than the scFv or Fab forms. To assess the binding of the selected antibodies to the MERS-CoV spike, 13 pairs of plasmids were co-transfected into HEK293T cells respectively and the supernatants containing mAbs were used. Six mAbs were confirmed to specifically bind to MERS-CoV Spike by the enzyme-linked immunosorbent assay (ELISA) (Supplementary Figure 2). We further expressed and purified each antibody in FreeStyle 293F cells. The purified antibodies were used to evaluate the neutralizing activity against cell entry with pseudotyped MERS-CoV. In addition to MERS-S111 (MERS-27), antibody MERS-S41 was able to inhibit pseudotyped MERS-CoV entry into susceptible Huh7 cells, with an IC_50_ of approximately 0.022 μg/mL (Figure 1B). Surface plasmon resonance (SPR) demonstrated that purified MERS-S41 could bind the spike with an affinity of approximately 4.6 nM (Figure 1C). MERS-S41 therefore has potent neutralizing activity and high affinity for the MERS-CoV spike glycoprotein.

### Structure determination

**FIGURE 2:**
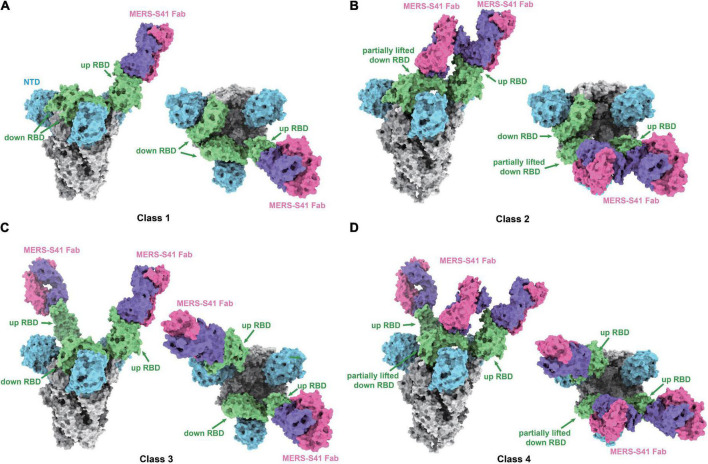
Cryo-EM structures of the MERS-S41 Fab bound to MERS-CoV spike trimer. **(A)** Class 1 shows MERS-S41 Fab bound to one “up” RBD. **(B)** Class 2 shows one “up” RBD bound by two MERS-S41 Fabs. **(C)** Class 3 shows two “up” RBDs bound by two MERS-S41 Fabs. **(D)** Class 4 shows two “up” RBDs bound by three MERS-S41 Fabs. In panels **(A–D)**, the MERS-CoV spike trimer NTDs are colored in blue, RBDs in green and other domains in gray. The light chain and heavy chain of MERS-S41 are colored in magenta and purple. The representations are models, shown as surface using Chimera X.

To determine the complex structure of MERS-S41 with the MERS-CoV spike trimer, we first digested recombinant MERS-S41 IgG to obtain the Fab (Supplementary Figure 1). We then incubated the MERS-S41 Fab with the S protein at a molar ratio of 3.6:1. The complex was visualized by cryo-EM, and the resulting images were processed using single-particle analysis methods (Supplementary Figures 3, 4 and Supplementary Table 2). The nominal resolution of the resolved spike trimer is 2.5Å (Supplementary Figure 4). To obtain better resolution of each RBD-Fab interaction, we performed focused three-dimensional classification and refinement. The resolution of each RBD-Fab ranged from 3.7 to 4.3Å, allowing us to build atomic models with sidechain accuracy (Supplementary Figure 4). In total, we identified four distinct classes (Figure 2; Supplementary Figure 3).

### MERS-S41 binds to different conformational states of the spike trimer

As we and others have previously demonstrated, coronavirus RBDs can sample “up” or “down” conformational states (Gui et al., 2017; Pallesen et al., 2017; Yuan et al., 2017). At least one RBD in the “up” position is necessary for the spike trimer to be in an activated, receptor-binding capable state, while all three RBDs in the “down” position keep the S protein in an inactive, receptor-binding incapable state (Song et al., 2018). The four different classes we identified showed that MERS-S41 bound to two distinct conformational states of the spike trimer (Figure 2). State 1 represented a conformation in which just one of the RBDs was “up” (42%) (Figures 2A,B), whereas State 2 corresponded to two “up” RBDs (58%) (Figures 2C,D). We did not observe MERS-S41 bound to spike trimers in which all RBDs were simultaneously “up” or “down.”

The MERS-S41 Fabs showed full saturation with one Fab bound to each “up” RBD (Figure 2) regardless of the state of the spike glycoprotein. This observation provided the first indication that MERS-S41 could potentially disrupt receptor binding (see below) by occupying the “up” RBDs required to bind DPP4 (Pallesen et al., 2017; Yuan et al., 2017). Besides binding to “up” RBDs, we also observed two classes in which MERS-S41 Fabs were bound to a partially lifted “down” RBD (Figures 2B,D). The Fab-bound “down” RBDs slightly lifted up with an additional angle of about 25.4° in class 2 and 27.7° in class 4 between the long axes (yellow lines) of the partially lifted “down” RBD and the horizontal plane, comparing to the corresponding non-Fab-bound “down” RBD in class 1 or class 3 (with an angle of about −13.7°) (Supplementary Figure 5). Notably, the partially lifted RBDs are incompatible with DPP4 binding (Supplementary Figures 5c,d). In this binding manner, MERS-S41 could potentially prevent further conformational changes to a fully activated, receptor-binding capable state with all three RBDs “up” rather than only inhibiting receptor binding.

### The MERS-S41 epitope overlaps with the DPP4 binding interface

**FIGURE 3:**
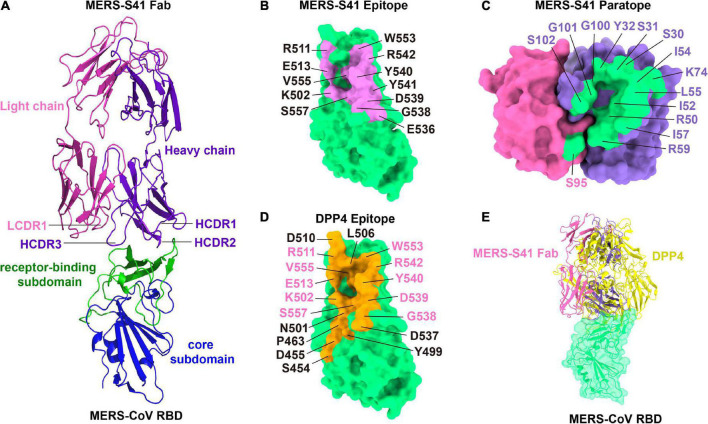
The binding interface of the MERS-S41 Fab and MERS-CoV RBD. **(A)** Structure of the MERS-S41 Fab bound to RBD. The RBD core subdomain is colored in blue, the receptor-binding subdomain in green, the MERS-S41 light chain in magenta, and the MERS-S41 heavy chain in purple. **(B)** The MERS-S41 epitope. The MERS-CoV RBD is shown in green surface with residues within 4Å of MERS-S41 Fab labeled and colored pink. **(C)** The MERS-S41 paratope. The MERS-S41 light and heavy chain surfaces are shown in magenta and purple, respectively. Residues within 4Å of the RBD are colored green and labeled in either magenta or purple, depending on whether they are from the light or heavy chain. **(D)** The DPP4 binding site. The MERS-CoV RBD is shown in green surface with residues within 4Å of DPP4 colored orange and labeled. Residues labeled in pink are also in the MERS-S41 epitope. **(E)** The RBD/MERS-S41 Fab structure superposed on the RBD-DPP4 structure (PDB 4L72). The MERS-CoV RBD is shown in green surface, DPP4 shown in yellow cartoon, and MERS-S41 Fab in pink cartoon.

The binding interface between MERS-S41 and the RBD consists of 12 residues from the RBD and 15 residues mainly from MERS-S41 heavy chain (Figure 3). Specifically, the RBD residues L506, Y540, R542, and W553 interact with S30, S31, and Y32 from the heavy chain HCDR1. The RBD residues K502, L506, E513, G538, D539, Y540, V555, and S557 interact with R50, I52, I54, L55, I57, and R59 from the heavy chain HCDR2. The RBD residues Y540, Y541, and R542 interact with G100, G101, and S102 from the heavy chain HCDR3. Residue K74 of the MERS-S41 heavy chain outside the CDRs interacts with residues L506, R511, and E513 of RBD. Only one residue S95 from the light chain LCDR1 interacts with E536 of RBD. Thus, a prominent feature of the interface is that recognition is mainly mediated by the heavy chain. Comparison of the MERS-S41 epitope with the DPP4 binding motif of RBD (Figures 3B,D) shows that there is an overlap with 10 of the 12 epitope residues, suggesting that MERS-S41 binds to the RBD from almost the same direction as DPP4. As expected, superposition of the RBD/MERS-S41 structure with the crystal structure of the MERS-CoV RBD in complex with DPP4 (PDB: 4L72) (Wang et al., 2013) showed severe steric clashes between the variable domain of the heavy chain and the β-propeller domain of DPP4 (Figure 3E). To further confirm that MERS-S41 can inhibit the binding of the spike trimer to DPP4, we performed a cell-surface staining assay by FACS. The results showed that MERS-S41 potently inhibited the binding of the spike trimer to Huh7 cells (Supplementary Figure 6).

### Neutralizing activity of MERS-S41 against pseudotyped MERS-CoV bearing naturally changing residues on the S glycoprotein

**FIGURE 4:**
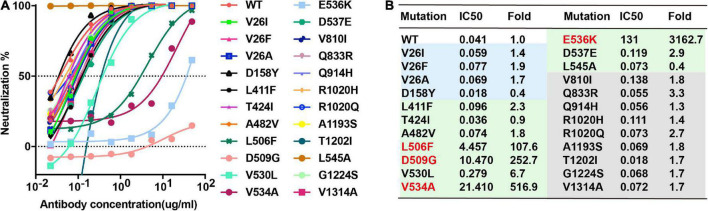
Breadth of MERS-S41 neutralization. **(A)** Neutralizing analysis of MERS-S41 IgG against MERS-CoV wild-type (WT) and its variant mutants. Site-directed mutations were introduced into the EMC strain to create 23 variant mutants according to natural mutations of MERS-CoV S. **(B)** Summary of MERS-S41 IgG mediated inhibition of infection by all pseudotyped viruses. IC_50_ neutralization titers for mutant EMC S variants are presented relative to wild-type S.

There are 22 natural variant mutants of the MERS-CoV EMC strain S glycoproteins and we have generated all the pseudotyped MERS-CoV EMC strain mutants (Zhou et al., 2019), including V26F, V26I, V26A, D158Y, L411F, T424I, A482Y, L506F, D509G, V530L, V534A, E536K, D537E, V810I, Q833R, Q914H, R1020H, R1020Q, A1193S, T1202I, G1224S, and V1314A. Among these mutations, we expected that E536K would enable MERS-CoV to escape neutralization by MERS-S41 as E536 is within the epitope. To confirm the binding and test its neutralizing activity against pseudotyped MERS-CoV bearing naturally changing residues, we performed the neutralizing analysis of MERS-S41 against MERS-CoV wild-type (WT) and its mutants. Indeed, E536K increased the IC_50_ value by more than 3000-fold and significantly reduced the activity of MERS-S41 (Figure 4). Three others RBD mutations, L506F, D509G and V534A, also increased the IC_50_ value by more than 100-fold (Figure 4). The results are consistent with the previous observation that MERS-CoV escaped the neutralization of RBD-targeting antibodies (Wang et al., 2015, 2018) when residue changes occurred on D506, D509, and E536.

## Discussion

Here, we have identified a MERS-CoV mAb, MERS-S41, that exhibits potent neutralizing activity. By combining cell surface staining assays with cryo-EM structural analysis of MERS-S41 in complex with the MERS-CoV spike trimer, we have demonstrated that MERS-S41 inhibits MERS-CoV entry by blocking binding to the receptor DPP4.

Based on the epitopes revealed by structural studies, we previously classified MERS-CoV RBD antibodies into three Groups (Xu et al., 2019). Group 1 and 2 antibodies directly compete with the receptor DPP4 but with different approach angles to the RBD, whereas Group 3 antibodies indirectly prevent DPP4 binding by inducing a conformation change of the RBD β5-β6 loop (Zhang et al., 2018) (Supplementary Figure 7). Group 2 antibodies typically have more potent neutralizing activity than those in Group 1 (Supplementary Table 3), as the approach angles of Group 2 antibodies are closer to that of DPP4. MERS-S41 binds an epitope almost fully overlapping with the receptor binding motif on the RBD (Figures 3B,D) and thus blocks attachment to DPP4 *via* direct competition. This observation explained its potent neutralizing activity and led us to classify it into the Group 2 MERS-CoV RBD antibodies (Supplementary Figure 7). MERS-S41 with IC_50_ at 0.053 μg/mL and K_*D*_ at 4.6 nM is stronger than most antibodies in Group 1 and at equal level with MERS-4 at Group 3. MERS-S41 is not as good as other antibodies among group 2, in terms of pseudoviruses neutralization activity and binding affinity. However, it is the one that was structurally analyzed with trimeric spike, not only just RBD domain. Of note, a structure of LCA60 in complex with trimeric spike was also determined, and it showed that LCA60 can bind both “up” and “down” RBDs in two states of spike. One state of spike was one “up” RBD and two “down” RBDs, another state was two “up” RBDs and one “down” RBD (Walls et al., 2019). Our MERS-S41 can bind to the MERS-CoV spike at different stoichiometries and the Fab-bound “down” RBDs were lifted up.

In our complex structures, MERS-S41 Fab can bind to “up” or partially lifted “down” RBDs, indicating that the neutralization mechanism is not only the direct competition with DPP4 but also potentially able to stop the conformation change of RBD from “down” to “up.” However, this speculated ability of trapping the RBD of S protein in the “down” conformation is limited. Based on our observations, MERS-S41 Fab binding to the non-“up” RBDs in the presence of excessive Fabs requires two conditions. First, MERS-S41 Fab only binds to the partially lifted “down” RBDs (Figure 2; Supplementary Figure 5). If we dock our Fab onto the “down” RBD in class 1 (Supplementary Figure 5a), class 2 (Supplementary Figure 8a), or class 3 (Supplementary Figure 5b), severe steric clashes occur. This analysis suggests that fully “down” RBDs are not accessible to MERS-S41. We therefore think that MERS-S41 recognizes an intermediate state between “down” and “up” although we cannot rule out that the Fab would induce this transformation. Second, the partially lifted “down” RBD requires an “up” RBD that sits at its pointing side (Figures 2B,D; Supplementary Figure 8a). The epitope of MERS-S41 is inaccessible in the inactivated state without “up” RBDs. We superimposed MERS-S41 Fab-RBD structure onto the structure of inactive MERS-CoV spike trimer (PDB 5W9J). Severe steric clashes would be expected between the MERS-S41 Fab and the “down” RBD of the neighboring S monomer (Supplementary Figure 8b).

## Materials and methods

### Cell lines

Vero E6, 293T, 293F, and Huh7 cell lines were bought from ATCC (Manassas, VA, USA) and cultured in Dulbecco’s Modified Eagle medium (DMEM) supplemented with 10% fetal bovine serum (FBS) and incubated at 37°C in a humidified atmosphere comprising 5% CO_2_.

### Protein expression and purification

The coding sequence of the MERS-CoV spike glycoprotein ectodomain (EMC strain, spike residues 1-1290) was ligated into the pFastBac-Dual vector (Invitrogen, Carlsbad, CA, USA) with a C-terminal T4 fibritin trimerization domain and a hexa-His-strep tap tag to facilitate purification. Briefly, the protein was expressed using the Bac-to-Bac baculovirus expression system and purified by sequentially applying Strep-Tactin and Superose 6 column (GE Healthcare, Chicago, IL, USA) with HBS buffer (10 mM HEPES, pH 7.2, 150 mM NaCl). Fractions containing MERS-CoV S glycoprotein were pooled and concentrated for subsequent biochemical analyses and EM studies.

The sequence encoding the MERS-S41 VL and VH were separately cloned into the backbone of antibody expression vectors containing the constant regions of human IgG1. The antibody MERS-S41 was expressed in FreeStyle 293-F cells by transient transfection and purified by affinity chromatography using Protein A Sepharose and size-exclusion chromatography. Purified MERS-S41 was exchanged into PBS and digested with papain protease (Sigma, St. Louis, MO, USA) overnight at 37°C. The digested antibody was then passed back over Protein A Sepharose to remove the Fc fragment, and the unbound Fab in the flow through was additionally purified using a Superdex 200 High Performance column (GE Healthcare).

### Selection of yeast library for MERS-CoV spike-specific scFvs

Human non-immune scFv library (∼1 × 10^9^), constructed from spleen and lymph node polyadenylated RNA pooled from 58 naïve humans, was provided by C. Baird (Pacific Northwest National Laboratory) (Feldhaus et al., 2003). Purified soluble S protein was used as a bait to select 2 × 10^9^ yeast cells by two rounds of MACS followed by three rounds of FACS with a BD FACSAria II sorter (San Jose, CA, USA). Between each round of selection, the sorted yeast cells were grown in SD-CAA and induced in SG-CAA medium as previously reported (Chao et al., 2006). After the second round of FACS, DNA plasmids were extracted from the sorted yeast population and transformed into *E. coli* DH5α for producing sufficient amounts of DNA for sequencing and sequence analysis.

The heavy and light chain genes of MERS-CoV spike–specific scFvs were separately cloned into backbone of antibody expression vectors containing the constant regions of IgG1. Whole-human IgG1 was expressed in 293T cells by transient transfection. The supernatants were serially diluted in PBS and applied on the IgG coated 96-well plates to confirm the IgG expression by a human IgG quantification kit (Abcam, Cambridge, UK). Then, MERS-CoV S glycoprotein at 1 μg/mL were used to coat plates overnight at 4°C, and the successfully expressed mAbs in each supernatant were serially diluted in PBS and assessed for binding affinity to the MERS-CoV spike by ELISA.

### Neutralizing assay of pseudotyped MERS-CoV

HEK293T cells cultured in 100 mm dish were co-transfected with 6 μg of pcDNA3.1-MERS-Spike-2p or its mutants and 24 μg of pNL4-3.luc.RE. The supernatants containing sufficient pseudotyped MERS-CoV were harvested 48–72 h post-transfection. Subsequently, the 50% tissue culture infectious dose (TCID_50_) was determined by infection of Huh7 cells. For the neutralization assay, 100 TCID_50_ per well of pseudotyped virus were incubated with 16 serial 1:3 dilutions of purified antibodies, Fabs or scFvs for 1 h at 37°C, after which Huh7 cells (about 1.5 × 10^4^ per well) were added. After incubation for 72 h at 37°C, the neutralizing activities of antibodies were determined by luciferase activity and presented as IC_50_, calculated using the dose-response inhibition function in GraphPad Prism 5 (GraphPad Software Inc.).

### Surface plasmon resonance experiments

Running buffer composed of 10 mM HEPES pH 7.2, 150 mM NaCl and 0.05% (v/v) Tween-20 was used during the analysis and all proteins were exchanged to the same buffer. The purified MERS-S41 IgG was covalently immobilized to a CM5 sensor chip (GE Healthcare) using Biacore T200 (GE Healthcare). The blank channel of the chip was used as the negative control. Serial dilutions of MERS-CoV Spike proteins were flowed through the chip sequentially. The resulting data were analyzed using Biacore T200 Evaluation Software 3.1 (GE Healthcare) by fitting to a 1:1 binding model.

### Florescence-activated cell sorting analysis of cell-surface staining

The binding between recombinant soluble MERS-CoV spike trimer (S) and human DPP4 expressed on the surface of Huh7 cells was measured using fluorescence-activated cell sorting (FACS). All cell-surface staining experiments were performed at room temperature. Soluble S protein with strep-tag (1 μg) was incubated with monoclonal antibodies (mAbs) in advance at molar ratios of 1:1, 1:3, 1:9, and 1:27 for 1 h. Huh7 cells were trypsinized and then incubated with S or S and mAbs mixtures for 1 h. After washing the un-bound S with PBS 3 times, the Huh7 cells were then stained with streptavidin APC (BD eBioscience, Franklin Lakes, NJ, USA) for another 45 min. Cells were subsequently washed with PBS 5 times and analyzed by flow cytometry on a FACS Aria III machine (BD eBiosciences).

### Cryoelectron microscopy data collection and image processing

Images for MERS-CoV spike ectodomains with MERS-S41 Fab were recorded using FEI Titan Krios microscope (Thermo Fisher Scientific, Waltham, MA, USA) operating at 300 kV with a Gatan K3 Summit direct electron detector (Gatan Inc., Pleasanton, CA, USA) at Tsinghua University. The automated software [AutoEMation2 (Scheres, 2012)] was used to collect 5,010 movies in super-resolution mode at a nominal magnification of 81,000× and at a defocus range between −1.5 and −2.0 μm. Each movie has a total accumulated exposure of 50 e^–^/Å^2^ fractionated in 32 frames of 175 ms exposure. The final image was binned 2-fold to a pixel size of 1.0825Å. Motion Correction (MotionCor2) (Zheng et al., 2017) and CTF-estimation (GCTF) (Zhang, 2016) were automatically executed by TsinghuaTitan.py program (developed by Dr. Fang Yang) during data collection. Data collection statistics are summarized in Supplementary Table 2.

The image processing procedures are presented in Supplementary Figure 3. Initially, 5,010 micrographs (3,360 Quantifoil micrographs and 1,650 Lacey carbon micrographs) were inspected and selected using the TsinghuaTitan.py program, followed by particle auto-picking using Gautomatch (developed by Kai Zhang^3^) or Relion 3.0 (Scheres, 2012; Zivanov et al., 2018). Multiple rounds of 2D classification were performed to eliminate bad particles, followed by 3D classification. 644,359 particles belonging to the best class were expanded with C3 symmetry, resulting in 1,933,007 particles, and followed by local 3D classification. Three classes had the same conformation but belonged to three different orientations around the C3 symmetry axis. Particles from the three classes were reorientated into the same orientation and duplicates removed, which yielded 424,969 particles. Refinement of these particles resulted in a map with a nominal resolution of 3.2Å. To improve the resolution further, CTF refinement, C3 symmetry and Bayesian Polishing were applied, which improved the overall resolution to 3.0, 2.8, and 2.5Å, successively. To improve the map density of RBD-Fab complex region, different masks of RBD-Fab complex were applied in focused 3D classification and subsequent refinement, which produced reconstructions of 4.3Å (RBD1-Fab, 118,198 particles), 4.2Å (RBD2-Fab, 88,100 particles), and 4.2Å (RBD3-Fab, 72,803 particles). Similarly, CTF refinement and Bayesian Polishing were applied to the RBD3-Fab complex, which improved the overall resolution to 3.7Å. To further classify different conformations, particle subtraction with a mask focused on all three RBD-Fab regions were applied, followed by 3D classification without alignment, which obtained four distinct conformations. Maps of the spike and three RBD-Fab regions were combined according to the classified distinct four classes to generate the final combined maps of the MERS-CoV spike-MERS-S41 complexes. All classification and refinement jobs were performed in Relion 3.0 or Relion 3.1. CTF refinement, Bayesian polishing, and particle subtraction were done in Relion 3.1.

### Model building and refinement

The atomic model of the MERS-CoV spike was built in Coot (Emsley et al., 2010) using PDB 5 × 58 as a starting model. The initial model of the MERS-S41 Fab was generated by SWISS-MODEL (Waterhouse et al., 2018) and fitted into the map using UCSF Chimera (Pettersen et al., 2004), followed by manual rebuilding in Coot. The atomic models were refined in real space using Phenix (Afonine et al., 2018), and validated using the Molprobity web application (Williams et al., 2018). UCSF Chimera and PyMol (Janson et al., 2017) were used for map segmentation and figure generation. Model refinement statistics are summarized in Supplementary Table 2.

### Figures

Figure panels depicting cryo-EM maps or atomic models were generated using Chimera (Pettersen et al., 2004) or ChimeraX (Pettersen et al., 2020). Maps colored by local resolution were generated using RELION 3.1 (Zivanov et al., 2018).

## Data availability statement

Publicly available datasets were analyzed in this study. This data can be found here: https://www.ncbi.nlm.nih.gov/igblast/.

## Author contributions

HZ and WJ isolated and characterized MERS-S41 and performed cell surface staining assays. SZ and HZ prepared proteins. SZ collected cryo-EM data and performed binding and neutralizing assays. JZ and SZ processed the cryo-EM data and built the model. HZ, LZ, and XW supervised the research. HZ and XW wrote the manuscript with input from SZ, WJ, JZ, ML, ZW, and LZ. All authors contributed to the article and approved the submitted version.

## Footnotes

1
https://covid19.who.int/

2
http://www.who.int/emergencies/mers-cov/en/

3
https://www2.mrc-lmb.cam.ac.uk/download/gautomatch-056/
