# Supplementary material for: Cryoelectron microscopy structures of a human neutralizing antibody bound to MERS-CoV spike glycoprotein
Source: Front Microbiol. 2022 Sep 28;13:988298. doi: 10.3389/fmicb.2022.988298 (PMC9554411; doi:10.3389/fmicb.2022.988298)
Supplement: Supplementary file 1 [file Data_Sheet_1.docx]

**Supplementary Data**


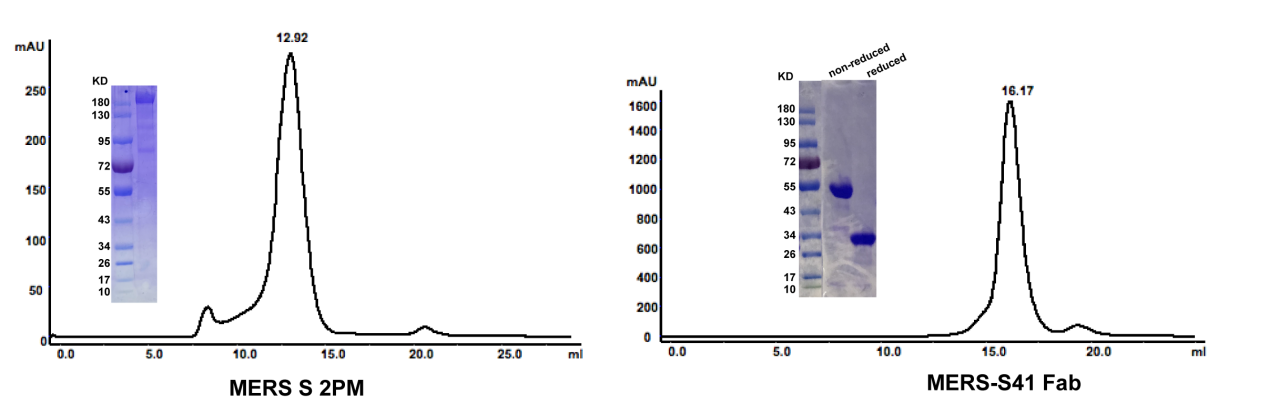


**Fig. S1. Antibody purification.** Elution profiles of the MERS-CoV S protein and MERS-S41 Fab from a size-exclusion column with SDS-PAGE gels inset.


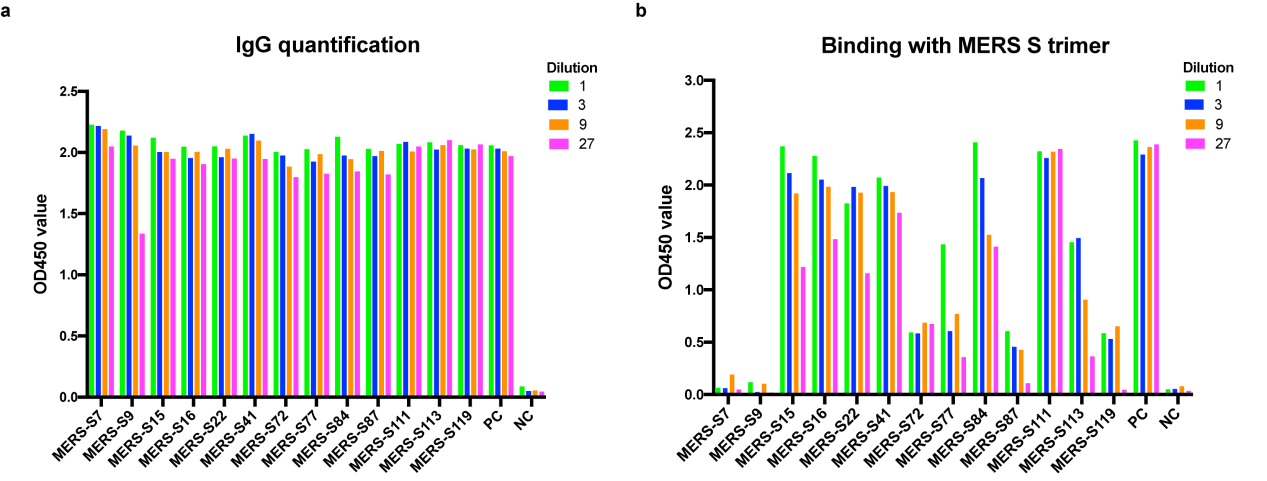


**Fig. S2** **The binding of isolated antibodies with MERS-CoV spike.** **a.** IgG quantification. The heavy and light chain genes of MERS-CoV spike–specific scFvs were cloned separately into backbone of antibody expression vectors containing the constant regions of IgG1. Whole-human IgG1 was expressed in HEK293T cells by transient transfection. The supernatants were serially diluted in PBS and applied on the IgG antibody coated 96-well plates to confirm the IgG expression by a human IgG quantification kit. b. Binding test by ELISA. Recombinant MERS-CoV S glycoprotein at 1 μg/mL were used to coat plates overnight at 4 °C, and the supernatant of each mAb including MERS-S41 were serially diluted in PBS and assessed for binding affinity to the MERS-CoV spike.


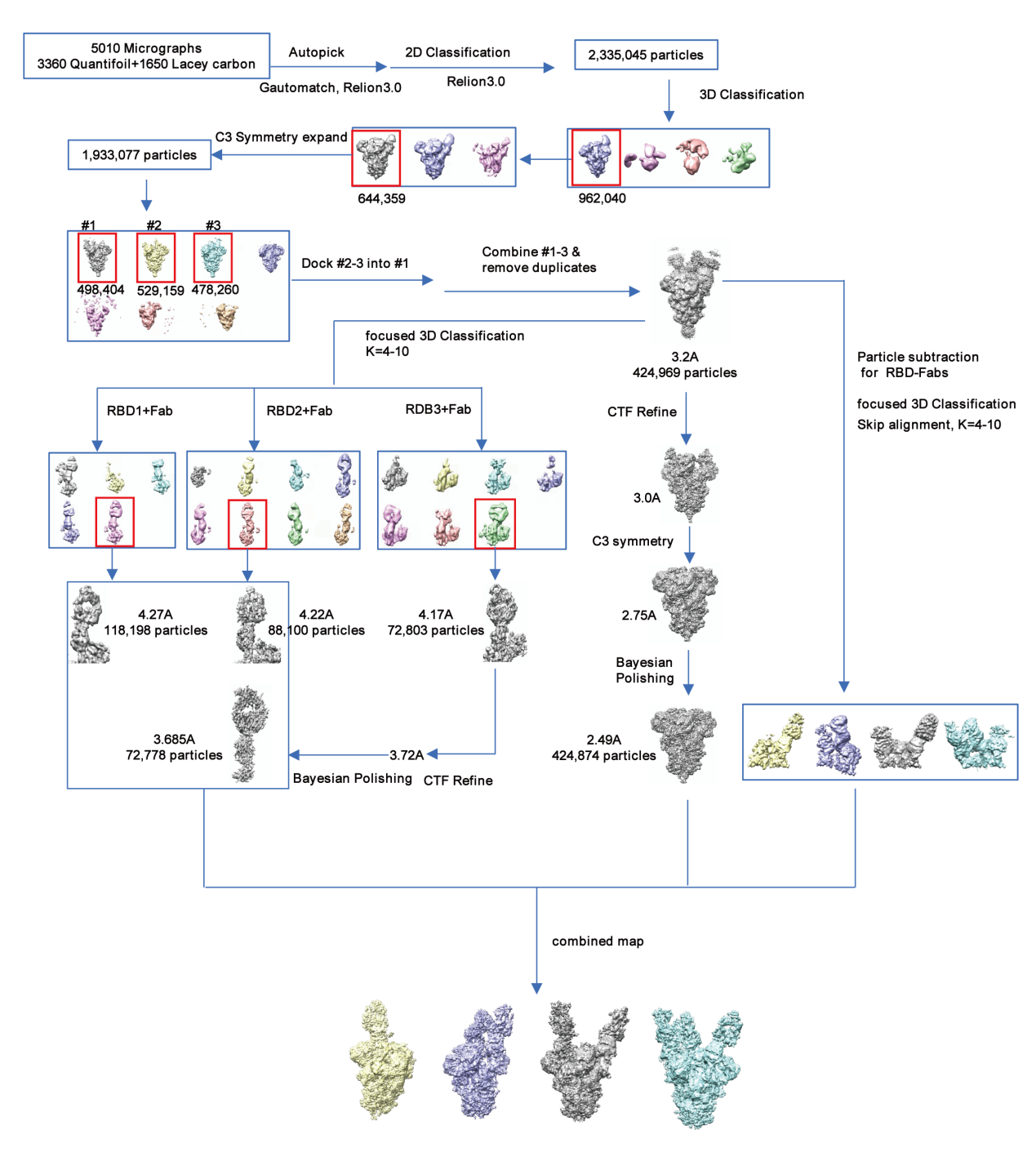


**Fig. S3 Cryo-EM data processing workflow.**


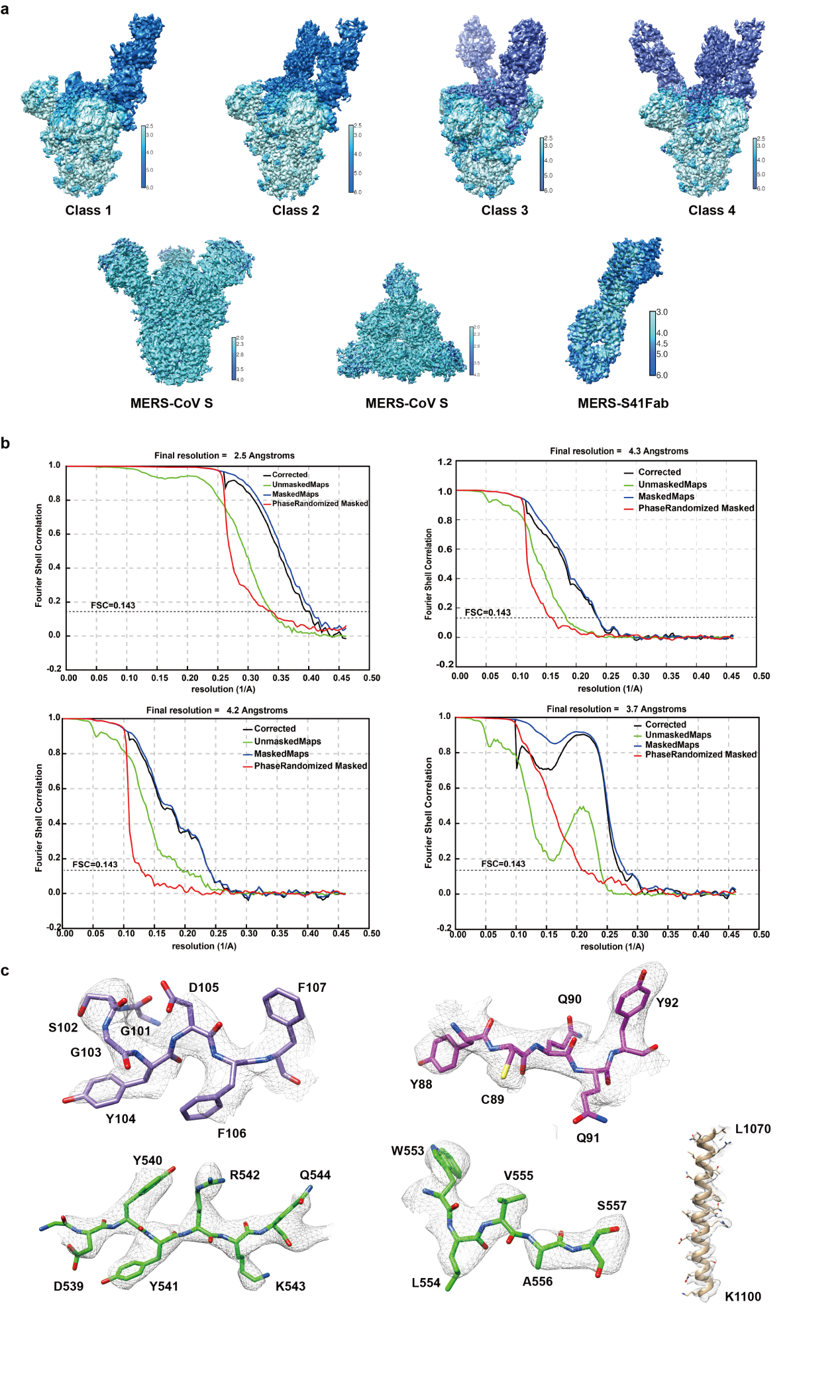


**Fig. S4 Cryo-EM structure validations. a,** Local resolution map of the structures of the MERS-S41 Fab bound with MERS-CoV spike, MERS-CoV spike glycoprotein (side view and top view) and MERS-S41Fab. The color scales indicate resolution. **b**, Fourier shell correlation (FSC) curve of the corrected, unmasked, masked and phase randomized cryo-EM reconstructions of the MERS-CoV spike glycoprotein, RBD1 with MERS-S41 Fab, RBD2 with MERS-S41 Fab, and RBD3 with MERS-S41 Fab. The resolution was estimated with the FSC=0.143 criterion. **c**, Representative densities from the MERS-S41 Fab heavy chain, light chain, MERS-CoV spike glycoprotein RBD and S2 region. The MERS-S41 Fab heavy chain, light chain, spike RBD and S2 are shown in purple, pink, green and brown, respectively.


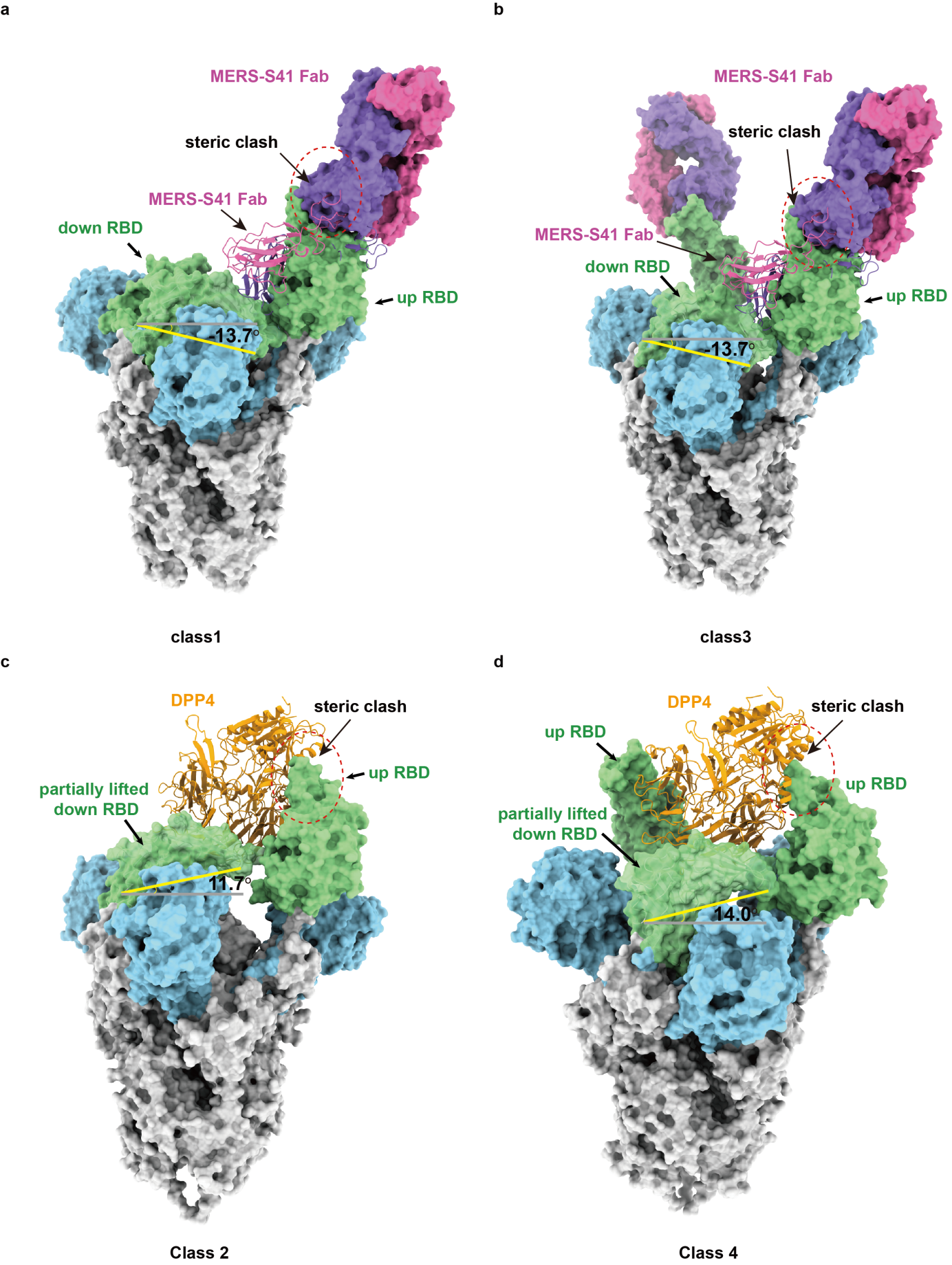


**Fig. S5 MERS-S41 Fab binds to partially lifted ‘down’ RBDs. a,b,** MERS-S41 Fab structures superimposed on the ‘down’ RBD of class 1 and class 3 structures. MERS-S41 Fab and neighboring ‘up’ RBD would clash upon binding to MERS-CoV S. The docked MERS-S41 Fab is shown in cartoon, with heavy chain in purple and light chain in pink. The angles between the long axes (yellow lines) of the ‘down’ RBD and the horizontal plane are shown. **c,d**, RBD-DPP4 structure (PDB: 4L72) superimposed on the partially lifted ‘down’ RBD of class 2 and class 4 structures. DPP4 and neighboring ‘up’ RBD would clash upon binding to MERS-CoV S. The docked DPP4 is shown in orange cartoon. The angles between the long axes (yellow lines) of the partially lifted ‘down’ RBD and the horizontal plane are shown. The MERS-S41 Fab structures are hidden in the class 2 and class 4 structures.


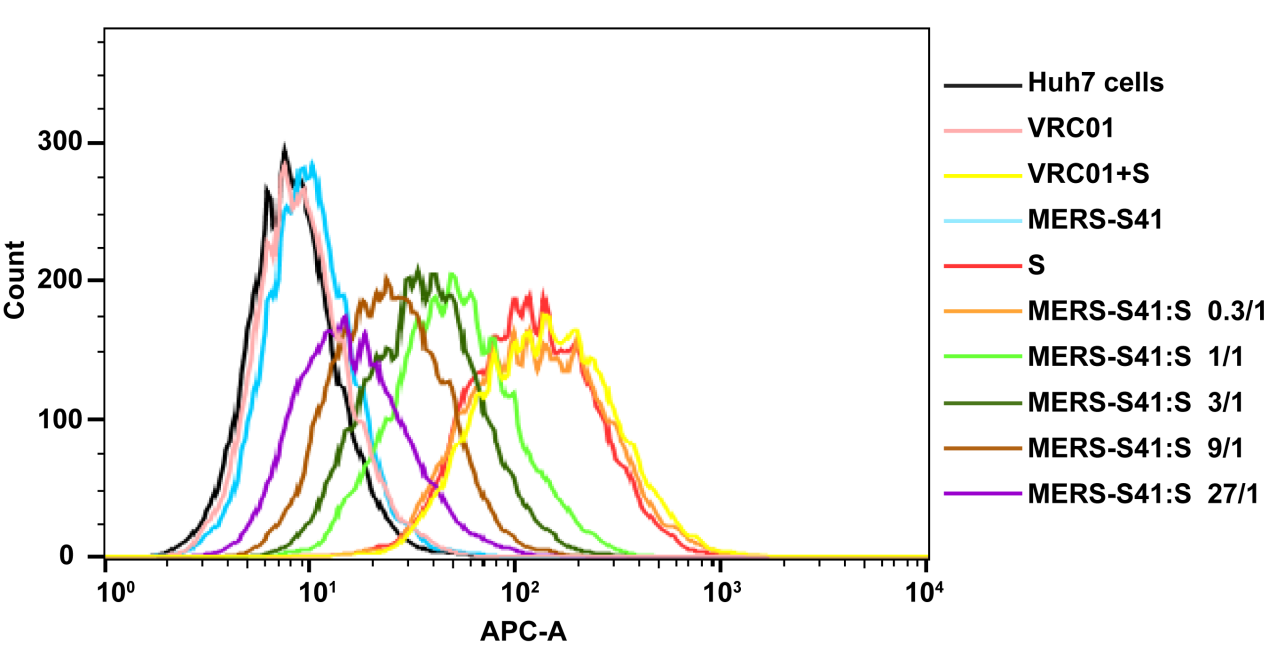


**Fig. S6 MERS-S41 inhibiting virus binding to its receptor DPP4.** Inhibition of the binding between recombinant soluble MERS-CoV spike trimer (S) and human DPP4 expressed on the Huh7 cell-surface. Soluble MERS-CoV spike trimer (S) with strep-tag (1 μg) was incubated with MERS-S41 IgG in advance at a molar ratio 1:0.3, 1:1, 1:3, 1:9, and 1:27 for 1 h. Huh7 cells were incubated with S or S and MERS-S41 IgG mixtures for 1 h. After washing the unbound S, Huh7 cells were stained with streptavidin APC and analyzed by fluorescence-activated cell sorting (FACS). The amounts of S-bound Huh-7 cells were measured and characterized as median fluorescence intensity.


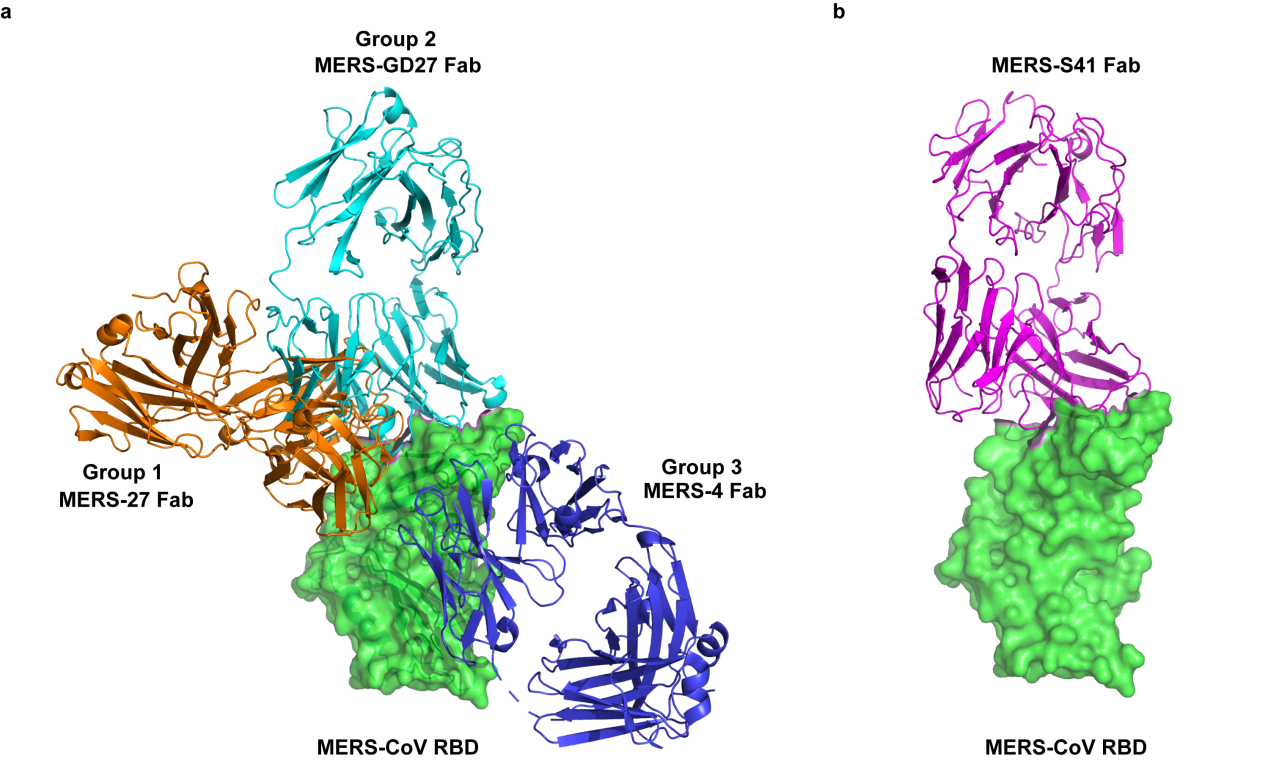


**Fig. S7** **Structures of MERS-CoV neutralizing antibodies targeting RBD.** **a,** Three group representative antibodies of MERS-CoV RBD. MERS-27 (PDB: 4ZS6) represents Group 1, MERS-GD27 represents Group 2 and MERS-4 (PDB: 5ZXV) represents Group 3. The MERS-CoV RBD is shown in green surface and antibodies shown as cartoon. **b**, MERS-S41 belong to Group 2 MERS-CoV RBD antibodies.


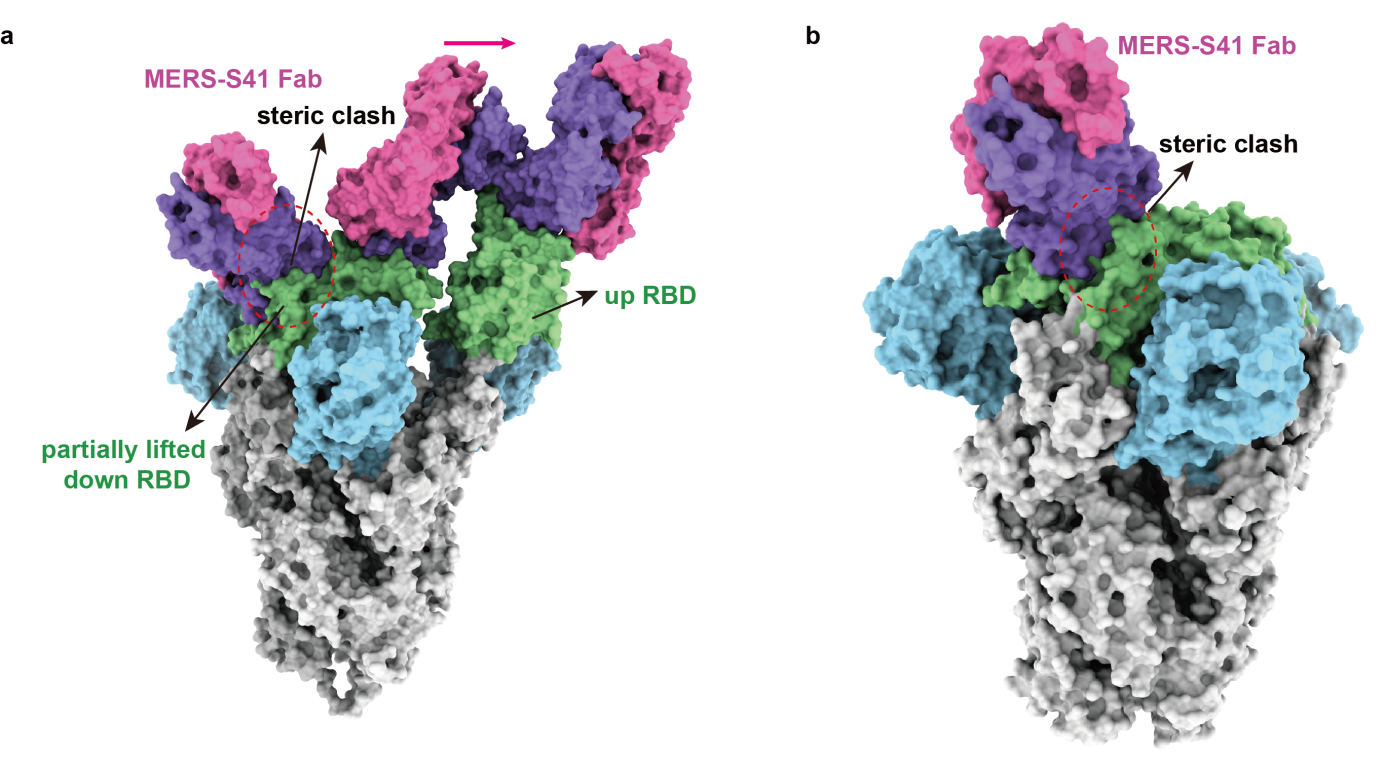


**Fig. S8 Steric clash between MERS-S41 Fab and MERS-CoV spike trimer. a,** Three MERS-S41 Fab/RBD structures superposed on the MERS-CoV spike trimer of Class 2 state. Steric clashes happened between the MERS-S41 Fab combined with the ‘down’ RBD and the partially lifted ‘down’ RBD of the neighboring S monomer. The pink arrow indicates pointing side of MERS-S41 Fab. **b,** One MERS-S41 Fab/RBD structure superposed on the closed MERS-CoV spike trimer (PDB 5W9J). Steric clashes happened between MERS-S41 Fab and the ‘down’ RBD of the neighboring S monomer.

**Tabel S1 Sequence characters of selected monoclonal antibodies.**


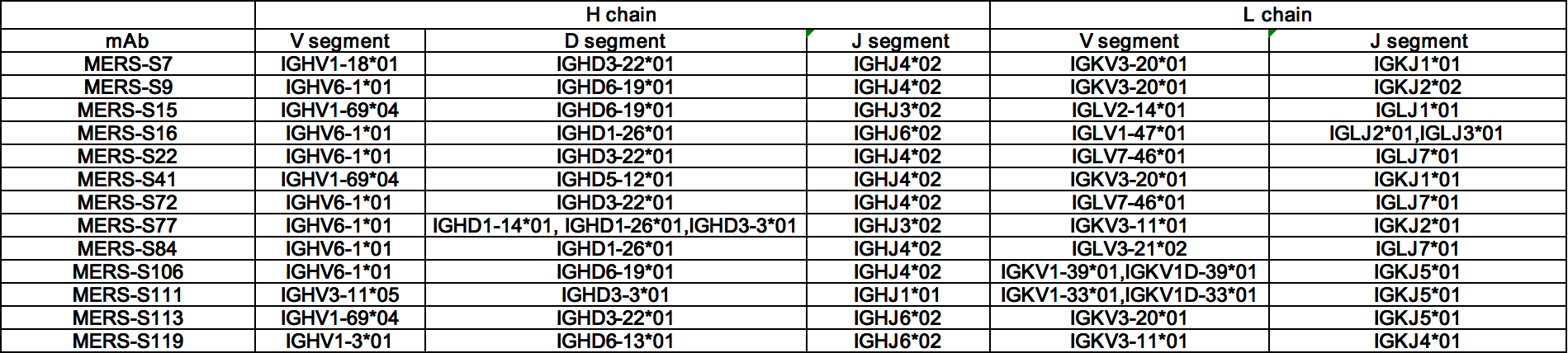


**Table S2 Cryo-EM data collection, refinement, and validation statistics**

| Data collection and processing | MERS-S41Fab-RBD  (PDB-7X26  EMDB-32959) | MERS-S41Fab-  spike Class1  (PDB-7X2A  EMDB-32963) | MERS-S41Fab-  spike Class2  (PDB-7X29  EMDB-32962) |
| --- | --- | --- | --- |
| Magnification | ×81000 | ×81000 | ×81000 |
| Voltage (kV) | 300 | 300 | 300 |
| Electron exposure (e^–^/Å^2^) | 50 | 50 | 50 |
| Defocus range (μm) | -1.5 to -2.0 | -1.5 to -2.0 | -1.5 to -2.0 |
| Pixel size (Å) | 1.0825 | 1.0825 | 1.0825 |
| Symmetry imposed | C1 | C1 | C1 |
| Initial particle images (no.) | 2,335,045 | 424,969 | 424,969 |
| Final particle images (no.) | 72,778 | 57,455 | 100,207 |
| Map resolution (Å) | 3.68 | 4.27 | 4.27 |
| FSC threshold | 0.143 | 0.143 | 0.143 |
| Map resolution range (Å) | 3.68-6 | 4.27-6 | 4.27-6 |
| Refinement |  |  |  |
| Initial model used (PDB code) | 5X58 | 5X58 | 5X58 |
| FSC threshold | 0.143 | 0.143 | 0.143 |
| Map sharpening B factor (Å^2^) | -70 | -198 | -198 |
| Model composition |  |  |  |
| Non-hydrogen atoms | 4837 | 30079 | 32452 |
| Protein residues | 640 | 3910 | 4230 |
| Ligands | NAG | 0 | 0 |
| B factors (Å^2^) |  |  |  |
| Protein | 104.84 | 135.11 | 135.98 |
| Ligand | 31.46 | 0 | 0 |
| R.m.s. deviations |  |  |  |
| Bond lengths (Å) | 0.006 | 0.025 | 0.014 |
| Bond angles (°) | 0.862 | 2.071 | 1.750 |
| Validation |  |  |  |
| MolProbity score | 2.34 | 2.55 | 2.33 |
| Clashscore | 15.38 | 14.86 | 7.36 |
| Poor rotamers (%) | 0.00 | 4.29 | 8.77 |
| Ramachandran plot |  |  |  |
| Favored (%) | 85.49 | 94.12 | 96.74 |
| Allowed (%) | 14.35 | 5.03 | 2.67 |
| Disallowed (%) | 0.16 | 0.85 | 0.60 |

| Data collection and processing | MERS-S41Fab-  spike Class3  (PDB-7X28  EMDB-32961) | MERS-S41Fab-  spike Class4  (PDB-7X25  EMDB-32958) |
| --- | --- | --- |
| Magnification | ×81000 | ×81000 |
| Voltage (kV) | 300 | 300 |
| Electron exposure (e^–^/Å^2^) | 50 | 50 |
| Defocus range (μm) | -1.5 to -2.0 | -1.5 to -2.0 |
| Pixel size (Å) | 1.0825 | 1.0825 |
| Symmetry imposed | C1 | C1 |
| Initial particle images (no.) | 424,969 | 424,969 |
| Final particle images (no.) | 118,566 | 97,317 |
| Map resolution (Å) | 4.27 | 4.27 |
| FSC threshold | 0.143 | 0.143 |
| Map resolution range (Å) | 4.27-6 | 4.27-6 |
| Refinement |  |  |
| Initial model used (PDB code) | 5X58 | 5X58 |
| FSC threshold | 0.143 | 0.143 |
| Map sharpening B factor (Å^2^) | -198 | -198 |
| Model composition |  |  |
| Non-hydrogen atoms | 31582 | 37110 |
| Protein residues | 4119 | 4844 |
| Ligands | 0 | 0 |
| B factors (Å^2^) |  |  |
| Protein | 105.61 | 135.98 |
| Ligand | 0 | 0 |
| R.m.s. deviations |  |  |
| Bond lengths (Å) | 0.013 | 0.008 |
| Bond angles (°) | 1.516 | 1.088 |
| Validation |  |  |
| MolProbity score | 2.27 | 2.78 |
| Clashscore | 7.64 | 8.43 |
| Poor rotamers (%) | 4.16 | 9.11 |
| Ramachandran plot |  |  |
| Favored (%) | 97.03 | 89.09 |
| Allowed (%) | 2.72 | 10.09 |
| Disallowed (%) | 0.25 | 0.82 |

| **Table S3 MERS-CoV monoclonal antibodies targeting RBD** | | |  | | |
| --- | --- | --- | --- | --- | --- |
| Name | Mechanism | Source | IC_50_ (μg/ml, pseudo) | K_d_ (nM, RBD) | Ref |
| MERS-27 | Group1 | Non-immune human ScFv (yeast library) | 9.21 | 71.2 | 1 |
| D12 |  | Mice immunized with S-DNA & S protein | 0.013 | 9.93 | 2 |
| 4C2h |  | Mice immunized with RBD | 1.8 | 217 | 3 |
| JC57-14 |  | vaccinated rhesus macaque | 0.0084 | N.A. | 4 |
| LCA60 |  | Human Survivor | 0.01 | 0.12 (S) | 5 |
| m336 | Group2 | Non-immune human ScFv (phage library) | 0.005 | 0.099 | 6, 7 |
| MCA1 |  | Human Survivor | 0.39 (live) | N.A. | 8 |
| CDC2-C2 |  | Human Survivor | 0.0057 | N.A. | 4 |
| MERS-GD27 |  | Human Survivor | 0.0010 | 0.775 (S) | 9 |
| MERS-S41 |  | Non-immune human ScFv (yeast library) | 0.022 | 4.6 (S) |  |
| MERS-4 | Group3 | Non-immune human ScFv (yeast library) | 0.0334 | 0.978 | 1 |

**References**

1 Jiang, L. et al. Potent neutralization of MERS-CoV by human neutralizing monoclonal antibodies to the viral spike glycoprotein. Sci Transl Med 6, 234ra259, doi:10.1126/scitranslmed.3008140 (2014).

2 Wang, L. et al. Evaluation of candidate vaccine approaches for MERS-CoV. Nat Commun 6, 7712, doi:10.1038/ncomms8712 (2015).

3 Li, Y. et al. A humanized neutralizing antibody against MERS-CoV targeting the receptor-binding domain of the spike protein. Cell Res 25, 1237-1249, doi:10.1038/cr.2015.113 (2015).

4 Wang, L. et al. Importance of Neutralizing Monoclonal Antibodies Targeting Multiple Antigenic Sites on the Middle East Respiratory Syndrome Coronavirus Spike Glycoprotein To Avoid Neutralization Escape. J Virol 92, doi:10.1128/JVI.02002-17 (2018).

5 Walls, A. C. et al. Unexpected Receptor Functional Mimicry Elucidates Activation of Coronavirus Fusion. Cell 176, 1026-1039 e1015, doi:10.1016/j.cell.2018.12.028 (2019).

6 Ying, T. et al. Exceptionally potent neutralization of Middle East respiratory syndrome coronavirus by human monoclonal antibodies. J Virol 88, 7796-7805, doi:10.1128/JVI.00912-14 (2014).

7 Ying, T. et al. Junctional and allele-specific residues are critical for MERS-CoV neutralization by an exceptionally potent germline-like antibody. Nat Commun 6, 8223, doi:10.1038/ncomms9223 (2015).

8 Chen, Z. et al. Human Neutralizing Monoclonal Antibody Inhibition of Middle East Respiratory Syndrome Coronavirus Replication in the Common Marmoset. J Infect Dis 215, 1807-1815, doi:10.1093/infdis/jix209 (2017).

9 Niu, P. et al. Ultrapotent Human Neutralizing Antibody Repertoires Against Middle East Respiratory Syndrome Coronavirus From a Recovered Patient. J Infect Dis 218, 1249-1260, doi:10.1093/infdis/jiy311 (2018).
